# PG-Priming Enhances Doxorubicin Influx to Trigger Necrotic and Autophagic Cell Death in Oral Squamous Cell Carcinoma

**DOI:** 10.3390/jcm7100375

**Published:** 2018-10-21

**Authors:** Shian-Ren Lin, Ching-Feng Weng

**Affiliations:** Department of Life Science and Institute of Biotechnology, National Dong Hwa University, Hualien 97401, Taiwan; d9813003@gms.ndhu.edu.tw

**Keywords:** prodigiosin, doxorubicin, priming, influx, autophagy

## Abstract

Synergistic effects between natural compounds and chemotherapy drugs are believed to have fewer side effects with equivalent efficacy. However, the synergistic potential of prodigiosin (PG) with doxorubicin (Dox) chemotherapy is still unknown. This study explores the synergistic mechanism of PG and Dox against oral squamous cell carcinoma (OSCC) cells. Three OSCC cell lines were treated with different PG/Dox combinatory schemes for cytotoxicity tests and were further investigated for cell death characteristics by cell cycle flow cytometry and autophagy/apoptosis marker labelling. When OSCC cells were pretreated with PG, the cytotoxicity of the subsequent Dox-treatment was 30% higher than Dox alone. The cytotoxic efficacy of PG-pretreated was found better than those of PG plus Dox co-treatment and Dox-pretreatment. Increase of Sub-G1 phase and caspase-3/LC-3 levels without poly (ADP-ribose) polymeras (PARP) elevation indicated both autophagy and necrosis occurred in OSCC cells. Dox flux after PG-priming was further evaluated by rhodamine-123 accumulation and Dox transporters analysis to elucidate the PG-priming effect. PG-priming autophagy enhanced Dox accumulation according to the increase of rhodamine-123 accumulation without the alterations of Dox transporters. Additionally, the cause of PG-triggered autophagy was determined by co-treatment with endoplasmic reticulum (ER) stress or AMP-activated protein kinase (AMPK) inhibitor. PG-induced autophagy was not related to nutrient deprivation and ER stress was proved by co-treatment with specific inhibitor. Taken together, PG-priming autophagy could sensitize OSCC cells by promoting Dox influx without regulation of Dox transporter. The PG-priming might be a promising adjuvant approach for the chemotherapy of OSCC.

## 1. Introduction

Doxorubicin (Adriamycin, Dox), isolated from soil bacteria *Streptomyces peucetius*, is the first member of anthracyclines (including daunorubicin, epirubicin, and idarubicin) [1,2]. The main action of Dox is intercalating within DNA pairs, which leads to the inhibition of topoisomerase IIβ and results in cell cycle blockage [3]. Rendering non-tissue specific characteristics, Dox gets wide indications including leukemia, neuroblastoma, breast carcinoma, ovarian carcinoma, and most recurrent or metastatic cancer [4]. Apart from these, the non-specific characteristics of Dox cause some severe adverse effects in cancer patients including immunosuppression, bone marrow suppression, hepatotoxicity, cardiotoxicity, and mucositis [5,6]. The cause of side effects is from the inhibition of cell division as well as reactive oxygen species (ROS) by-products (doxorubicin-semiquinone, doxorubicinol, dexrazoxane, and 7-deoxy-doxorubicinone) during metabolism of doxorubicin in mitochondria [2,7,8,9]. As a result, these adverse effects might limit the applicable dosage and cancer treating efficacy of Dox. Accordingly, the alternative approach or new formulation to attenuate Dox side effects and enhance Dox efficacy turns out to be a crucial issue for Dox use in the regimen of chemotherapy. Recently, nanocarriers have exerted a favorable theme and some research has focused on this topic to dissolve these obstacles, such as Dox encapsulated in pH-sensitive, ultrasonic-responsive, or co-capsulated with MDR-1 inhibitor in PEGylated, liposome, or PLGA nano-carrier, which also promote Dox uptake [10,11,12,13,14]. However, cytotoxicity of nanoparticle conjugated Dox was 10 times lower than free-form Dox, which also restricted the use in cancer treatment [15].

A long treatment period with a low dose chemotherapeutic drug might induce chemoresistance within cancer cells and subsequently toxicity could affect its use [16]. Notably, prevailing mechanisms of chemoresistance could be classified into the following seven phases: drug flux, DNA damage repair, cell death inhibition, epithelial-mesenchymal transition (EMT), drug target alteration, drug inactivation, and epigenetics [17]. In Dox resistance, dug efflux would be the most concerning phase [16]. Dox can import into cells through solute carrier family 22 member 16 (SLC22A16, also known organic cation transporter 6, oct-6) and export by ATP-binding cassette transporter family members, in which multidrug-related protein 1 (MDR-1 or p-glycoprotein) and breast cancer resistance protein (BCRP or ABCG2) are involved [3]. These proteins will be regulated (upregulation of exporter and downregulation of importer) during long-term exposure to a non-toxic dose of Dox [18]. Thereby, numerous studies have put more attention to reducing MDR over-expression to reverse multidrug resistance. CRISPER/Cas-9 gene editing, ursodeoxycholic acid, or *Zingiber officinale* Roscoe, have been reported to down-regulate *ABCB1* gene expressions in chemo-resistant cancer cells [19,20,21].

Prodigiosin (PG, PubChem CID: 5351169) is a red prodiginine pigment isolated from various bacteria including *Serratia marcescens, Pseudoalteromonas rubra, Hahella chejuensis,* and actinomycete bacteria [22,23,24,25]. Even though the original biological function in producer bacteria remains unclear, PG has been identified with numerous biological activities including antimicrobial [26,27,28,29], antimalarial [26,27,30], and antitumor [26,27,31,32,33,34] activities. Moreover, PG showed apoptotic inducing property in many cancer types such as lung cancer [35,36,37], breast cancer [38,39], colorectal cancer [40,41,42], leukemia [43,44], and hepatocellular carcinoma [45] without normal cell cytotoxicity [41,46]. Recently, PG has also been identified as an autophagy inducer in OSCC cells [47,48]. However, the application of PG as an adjuvant in chemotherapy is still unknown.

## 2. Experimental Section

### 2.1. Research Aims

This study was conducted to explore the potential of PG combined with doxorubicin in anti-cancer activity by using oral squamous cell carcinoma (OSCC) cells as a test platform. Next, experiments tested the synergistic effects of PG and Dox against OSCC cells to evaluate the adjuvant potential of PG for cancer therapy. Furthermore, the underlying molecular mechanisms of enhanced doxorubicin cytotoxicity under PG-priming were also investigated.

### 2.2. Reagents

Cell-cultured medium and reagents were purchased from Thermo-Fisher (Waltham, MA, USA). Prodigiosin was purified by Dr. Yu-Hsin Chen (Department of Life Science, National Dong-Hwa University, Hualien, Taiwan). Liposome-coated doxorubicin (abbreviated as Dox) was obtained from Dr. Ming-Fang Cheng (Division of Histology and Clinical Pathology, Hualian Army Forces General Hospital, Hualien, Taiwan). Inhibitors used in this study were purchased from Santa Cruz Biotechnology (Dallas, TX, USA). General chemicals were purchased from Sigma Aldrich (Merck KGaA, Darmstadt, Germany). Polyvinylidene difluoride (PVDF) membrane used in Western blotting was obtained from GE Healthcare (Chicago, IL, USA). The antibodies used in this study were obtained from Santa Cruz Biotechnology, as shown in Table 1.

### 2.3. Cell Culture

Cell lines used in this study were obtained from different sources: Human pharynx squamous carcinoma FaDu from Dr. Chun-Shu Lin (Radiation Oncology Department, Tri-Service General Hospital, Taipei, Taiwan), human oral squamous cell carcinoma cell line OECM1 and tongue carcinoma cell line SAS from Professor Ta-Chun Yuan (Department of Life Science, National Dong Hwa University, Hualien, Taiwan), and human bronchus epithelial cell BEAS-2b from American Type Culture Collection (ATCC). OECM1 and SAS were cultured in Roswell Park Memorial Institute medium 1640 (RPMI 1640), FaDu in minimum essential medium (MEM), and BEAS-2b in Dulbecco’s Modified Eagle Medium (DMEM) and medium was changed every 2 days. All culture media were mixed with 10% fetal bovine serum (FBS) and 1% antibiotic-antimycotic and cultured within 37 °C, 5% CO_2_ incubator (Thermo-Fisher). Cells were detached by 0.25% trypsin/ethylenediaminetetraacetic acid (EDTA) for further experiments. All experiments were obtained within 20 passages concerning uniformity and reproducibility.

### 2.4. Cytotoxicity Assay

Cytotoxicity was determined using a colorimetric assay by MTT (3-(4,5-dimethylthiazol-2-yl)-2,5-diphenyltetrazolium bromide) previously described in the literature [48]. The optical density (OD) alteration of mitochondrial enzymatic activity was converted into the cell numbers according to the cell viability or cytotoxicity. Briefly, 1 × 10^4^ cells per wells were seeded in 96-well plate and incubated in culture conditions overnight. Then, cells were divided into the six following treatment groups: (1) PG-Dox group: treated with PG followed by Dox, (2) Dox-PG group treated with Dox and then PG, and (3) PG + Dox group: treated PG and Dox at the same time, respectively. An additional three groups performed the same treatments with the above-described and replaced Dox with cisplatin. All treatments were carried out in 12 h and 1 mg/mL of MTT solution was added and further incubated for 4 h at 37 °C as treatment finished. Finally, liquid in wells was replaced by dimethyl sulfoxide (DMSO), and the absorbance at 570 nm was measured by Multiskan™ FC microplate photometer (Thermo-Fisher). Cytotoxicity of each treatment was represented by cell viability which calculated from the absorbance ratio at 570 nm between treated and untreated groups.

To understand the cause of PG- and Dox-induced cell death, inhibitor recovering assays were also performed following the above protocol. Autophagy inhibitors (bafilomycin A1 and 3-methyladenine), endoplasmic reticulum (ER) stress inhibitors (tauroursodeoxycholic acid, TUDC), and AMPK inhibitors (dorsomorphin, CC) were cotreated with various concentrations of PG or Dox, respectively.

### 2.5. Cell Cycle Analysis

Cell cycle analysis was carried out by flow cytometry. Firstly, 1 × 10^6^ cells/well of OSCC were seeded into 6-well plates and incubated in culture condition overnight. To understand drug-pretreated effect, cells were treated with PG for 12 h, Dox for 12 h, or PG for 12 h followed by Dox for additional 12 h, respectively. After treatment, cells including culture medium were collected using trypsin/EDTA and washed by phosphate buffer saline (PBS) twice before being fixed with pre-cooled 70% ethanol/PBS overnight. After fixation, cells were washed twice by PBS and stained with staining buffer (20 μg/mL of propidium iodide, 0.1% Triton X-100, 0.2 mg/mL RNase A) at 37 °C for 1 h. The fluorescent intensity in the cells was measured by a flow cytometer (Cytomics^TM^ FC500, Beckman, Fullerton, CA, USA). Data from 10^4^ cells were collected for each data file. Fluorescent intensities for each cell line were acquiesced and plotted by flow cytometer software. The gating of each phase was based on the acquisition histogram of untreated controls. Phases of each group were collected and the average of each phase was calculated within the groups.

### 2.6. Doxorubicin Flux Analysis

Efflux and influx of Dox was determined by indirect method which used rhodamine 123, a fluorescent Dox transporter substrate, detected as an indicator described previously [49,50]. Briefly, 1 × 10^4^ cells/well of OSCC were seeded in 96-well plate and incubated in culture condition overnight. After cell confluence, cells were divided into four groups and then treated with various regimens, respectively. For Rhodamine-influx: short-term influx assay: 0.5 μM of PG in full-culture medium was added and incubated for 1 h and replaced culture medium with 2 μM of rhodamine 123 in PBS for additional 1 h; long-term influx assay: the same treatment as short-term influx assay instead the incubation duration of PG from 1 h to 12 h. For Rhodamine-efflux: short-term efflux assay: 2 μM of rhodamine 123 in PBS was firstly incubated for 1 h and followed by 0.5 μM of PG in full-culture medium for additional 1 h; long-term efflux assay: the same as treatment as short-term influx assay instead the incubation duration of PG from 1 h to 12 h. After incubation, cells were trebly washed by PBS and lysed with 0.1% triton X-100 and the fluorescent intensity was determined at 485/538 nm by α-screen multi-plate reader (Perkin Elmer, Waltham, MA, USA). These rhodamine flux studies were used to estimate DOX flux.

### 2.7. Western Blotting

The detail protocol of Western blotting was described in our previous study [51]. In brief, 1 × 10^6^ cells/well of OSCC cells were seeded into a 6-well plate and treated the same as with cell cycle analysis. Cells were washed by PBS and lysed by radioimmunoprecipitation assay buffer (RIPA). Then, 30 μg of total proteins from cell lysates were electrophoretically separated by sodium dodecyl sulfate polyacrylamide gel electrophoresis (SDS-PAGE) and transferred into polyvinylidene difluoride (PVDF) membrane. Proteins of interest were identified via incubation with appropriate primary followed by horseradish peroxidase (HRP)-conjugated secondary antibodies and exposed to the iBright imaging system (Thermo-Fisher) for monitoring intensity of signals after soaking in enhance chemiluminescent (ECL) reagents. Data acquisition was also performed by the iBright imaging system, and signal intensity was normalized with GAPDH as an internal control.

### 2.8. Statistical Analysis

All quantified results were shown as mean ± standard deviation (SD) of three independent experiments. Significant analysis used a one-way ANOVA, followed by Dunnett’s test. A data histogram was built by GraphPad Prism 7.04 (La Jolla, CA, USA).

## 3. Results

### 3.1. Cytotoxicity Change of PG/Dox Treated Strategies

**Figure 1:**
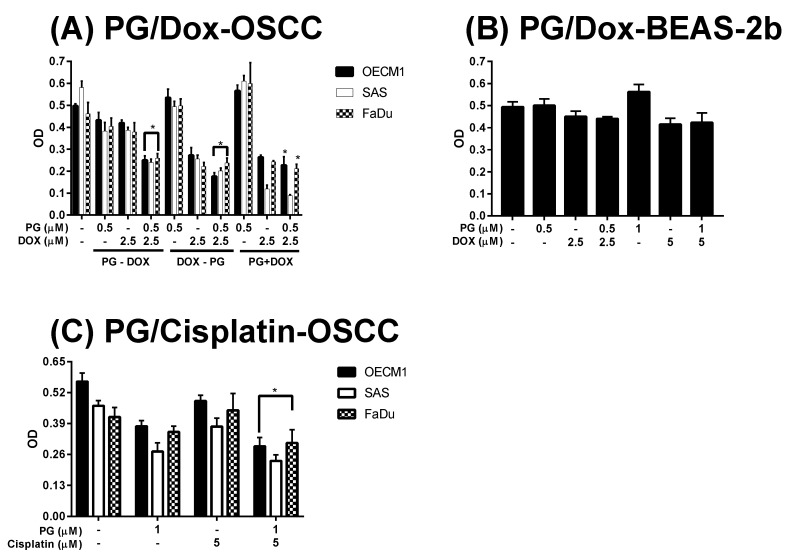
Alteration of cytotoxicity in sequential PG (prodigiosin)/Dox (doxorubicin) and PG/cisplatin combination in oral squamous cell carcinoma (OSCC) and BEAS-2b cells. (**A**) OSCC and (**B**) normal bronchus cells-BEAS-2b were treated with various schemes of PG and Dox, and (**C**) Cisplatin substituted Dox for 12 h and analyzed cell viability by 3-(4, 5-dimethylthiazol-2-yl)-2, 5-diphenyltetrazolium bromide (MTT) assay. The results were represented as mean ± SD from three individual experiments. * *p* < 0.05 as compared with Dox alone.

In this study, three combined manners (pretreatment, cotreatment, and posttreatment) of PG/Dox were tested in OSCC. In pretreatment and post-treatment approaches, chemicals were previously treated for 12 h and subsequently washed out, followed by new chemical treatment for additional 12 h. Therefore, the term “PG-pretreatment” would be defined as a “PG-priming” procedure in the subsequent section. The cell viability of all tested OSCC cells were declined in all combined strategies except cotreatment in SAS (*p* < 0.05). In three combined strategies, PG-pretreatment got the highest reducing levels (as compared with Dox alone) than those of the other two strategies, as shown in Figure 1A. This result posed the potential of PG-pretreatment as PG-priming in OSCC. When doubling the concentrations of PG, cell viability was the same as that of single concentration, revealed 0.5 μM of PG, which was the maximum concentration for PG-priming. Also, extending the PG-priming period up to 24 h, the cytotoxicity of Dox failed to exhibit an additive potentiation. These results indicated that 12 h of PG-priming might reach the maximum effect (data not shown). Moreover, with PG-priming in normal cell lines BEAS-2b, the cell viability of Dox treatment did not show the decrease as much as OSCC, even though the concentrations of PG and Dox were twice higher than that of OSCC, as shown in Figure 1B. This result indicated that PG-priming was more effective and less toxic than that of Dox alone. An additional experiment was to investigate whether the PG-priming effect could also be observed in golden chemotherapy drug cisplatin, however, the cytotoxic enhancement in PG/Dox combination could not be found in PG/cisplatin combination, as shown in Figure 1C. Taking all results together, PG-priming could enhance Dox cytotoxicity in OSCC cells through a Dox-related mechanism. In subsequent experiments, the type of cell death triggered by PG-priming and the underlying mechanism were further investigated.

### 3.2. Identification of Cell Death Characteristics

**Figure 2:**
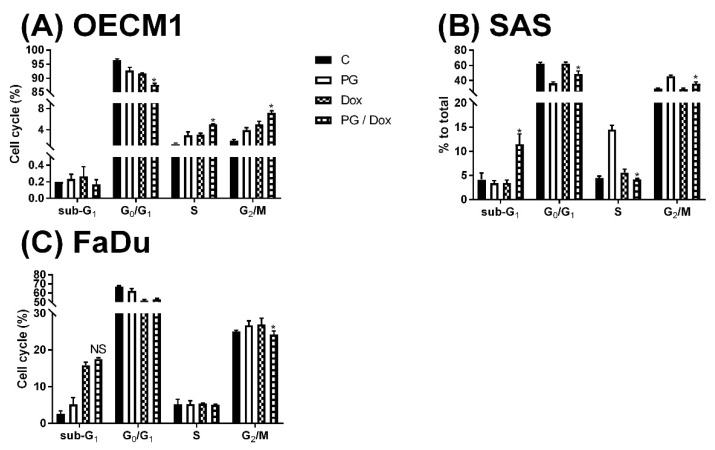
Alteration of cell cycle in (**A**) OECM1, (**B**) SAS, and (**C**) FaDu cells. OSCC cells were treated with PG/Dox for 12/12 h prior to staining with propidium iodide (PI) and fluorescent intensity was analyzed by flow cytometry. The results were represented as mean ± SD from three individual experiments. * *p* < 0.05 as compared with DOX alone.

**Figure 3:**
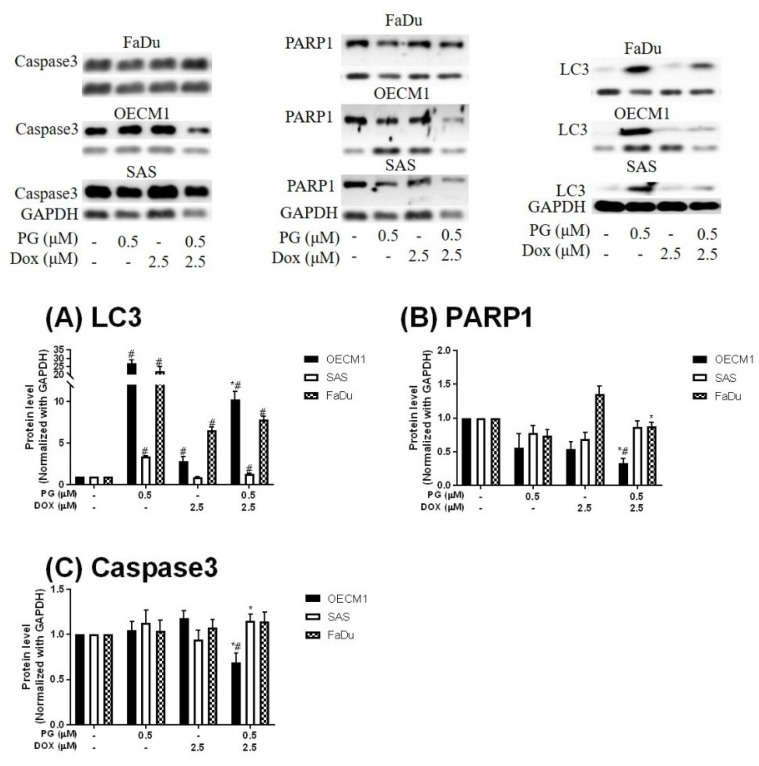
Expression of (**A**) LC3, (**B**) PARP1, and (**C**) Caspase3 in OSCCs after PG-priming. OSCC cells were treated with PG/Dox for 12/12 h and then desired protein levels were analyzed by Western blotting. The results were normalized with GAPDH and represented as mean ± SD from three individual experiments. Molecular weight: PARP1, 116 KDa; GAPDH, 37 KDa; Caspase3, 34 KDa; LC3, 18 KDa. # *p* < 0.05 compared with untreated control; * *p* < 0.05 as compared with Dox alone.

**Figure 4:**
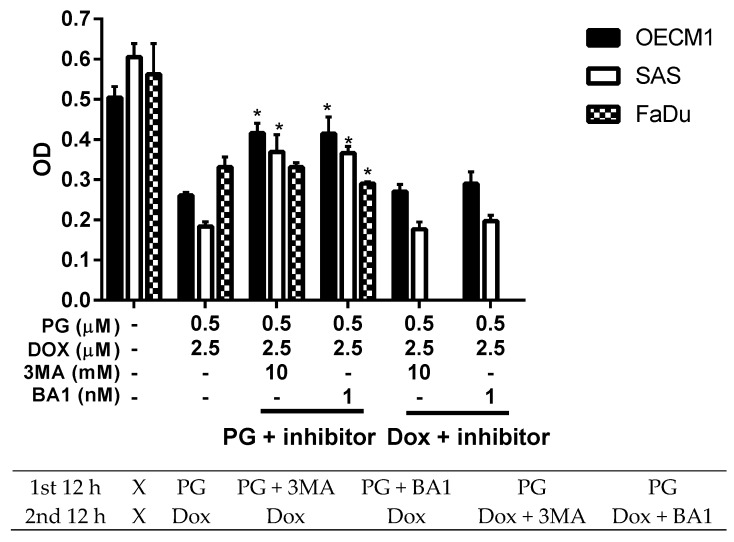
**Alteration of cell viability combined with autophagy inhibitor.** OSCC cells were treated with PG + inhibitor/DOX or PG/Dox + inhibitor and cell viability was analyzed. Table under figure was the scheme of treatment. “X” meant incubated with complete medium without PG or Dox. The results were represented as mean ± SD from three individual experiments. * *p* < 0.05 as compared with PG/Dox.

Numerous types of cell death were found in cells, but the most common types were apoptosis and autophagy, respectively. These two cell-death types could be distinguished by analyzing apoptosis and autophagy-related protein and cell markers, and the most obvious marker would be cell cycle analysis. In cell cycle analysis of SAS, Sub-G_1_ was significantly increased, but non-cleaved PARP1 and caspase-3 protein levels were not decreased after PG-priming, as shown in Figure 2B and Figure 3B,C. These results revealed that PG-priming prior to Dox treatment would lead to SAS undergoing necrosis. Moreover, while PG combined with autophagic inhibitor (bafilomycin A1 (BA1) and 3-methyladenine (3MA), cell viability of PG-priming could be recovered, as shown in Figure 4. This phenomenon might indicate that both necrosis and autophagy were activated after PG-priming and the autophagy would be a major clue, which was further confirmed by increases in LC3 protein levels, as shown in Figure 3A. In FaDu cells, Sub-G_1_ phase was not significantly increased after PG/Dox treatment, as shown in Figure 2C. Also, non-cleaved PARP1 and caspase-3 protein levels while PG-priming followed by Dox treatment were decreased when compared with Dox alone, as shown in Figure 3B,C. The results also showed the necrosis activation within FaDu cells similar to SAS cells. Unlike SAS cells, this cell viability could not be recovered by autophagy inhibitor, as shown in Figure 4. In FaDu cells, LC3 protein levels were also significantly increased in the PG/Dox group, as shown in Figure 3A. These results revealed that both necrosis and autophagy were activated in FaDu cells and necrosis would be the main cause of cytotoxicity, whereas OECM1 cells showed different patterns from the above two cells lines. The sub-G1 phase did not significantly increase either in the non-cleaved PARP1 or caspase-3 protein levels, as shown in Figure 2A and Figure 3B,C. Taken together, PG-priming cell death of OECM1 was not related to apoptosis or necrosis. On the other hand, the cell viability of OECM1 after PG-priming could be recovered by autophagy inhibitors, as shown in Figure 4. Also, LC3 protein levels were increased 10-fold, as shown in Figure 3A. These two results gave a clear clue for autophagy in OECM1 by PG-priming. Considering all above results, induced cell death characteristics in three different cell lines by PG-priming illustrated in different configurations. OECM1 showed autophagy, and SAS and FaDu posed both cell death and autophagy. In the subsequent experiments, the potential pathways of PG-enhanced Dox cytotoxicity were under investigation.

### 3.3. Doxorubicin Flux Affected by PG-Induced Autophagy

**Figure 5:**
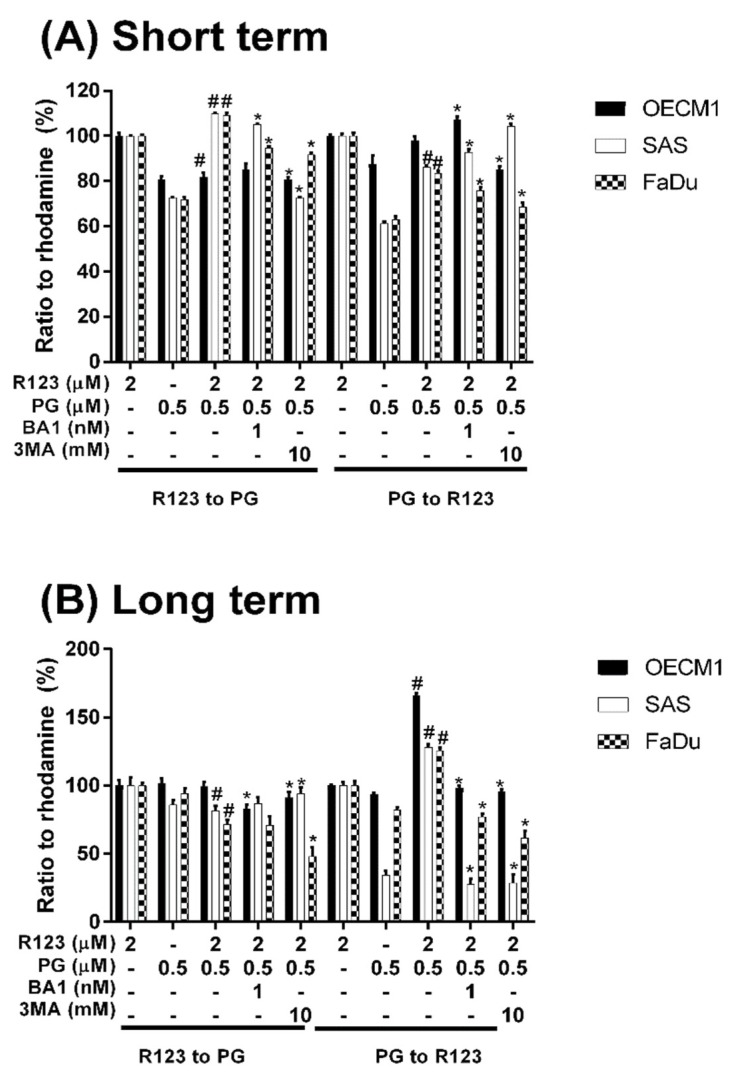
**Rhodamine 123 accumulation after PG pretreatment.** OSCC cells were treated with PG/R123 for (**A**) short term (1/1 h) and (**B**) long term (12/1 h) followed by analyzed fluorescent intensity within cells. The results were represented as mean ± SD from three individual experiments. # *p* < 0.05 as compared with R123 alone; * *p* < 0.05 compared with PG/R123 combination.

**Figure 6:**
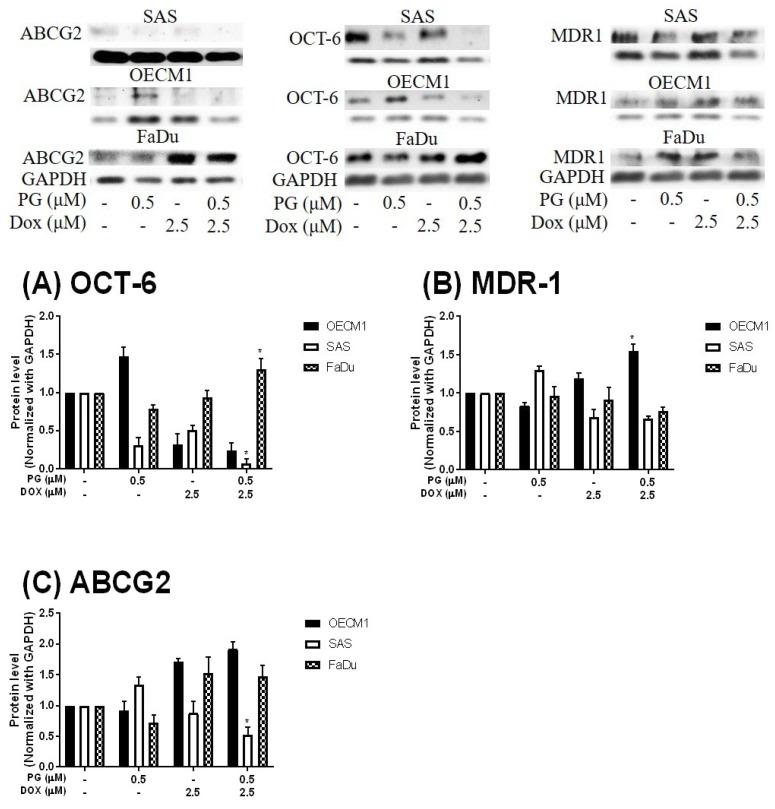
**Protein levels of Doxorubicin-related importer and exporter after PG-priming.** OSCC cells were treated with PG/Dox for 12/12 h and then were analyzed for Importer OCT-6 (**A**) and exporter MDR-1 (**B**) and ABCG2 (**C**) protein levels by Western blotting. The results were normalized with GAPDH and represented as mean ± SD from three individual experiments. Molecular weight: MDR-1, 170 KDa; ABCG2, 72 KDa; OCT-6, 58 KDa; GAPDH, 37 KDa. * *p* < 0.05 compared with Dox alone.

To measure the possible action of PG-induced autophagy, we examined the Dox flux in PG-priming OSCC cells. This Dox flux was determined by rhodamine-123 (R123) accumulation. R123 is a green fluorescent dye which acts as a Dox-transporter substrate over decades [52]. Accordingly, PG and Dox are all red fluorescence and PG is stronger fluorescence than that of Dox. After PG/Dox combination treatment, PG will interfere with the measurement of Dox fluorescent-intensity. Also, the less cytotoxic nature of R123 could eliminate the interference of cell death caused by Dox. Therefore, R123 was employed as an indicator to indirectly determine the Dox-flux in this study. In short-term priming (1 h), PG-priming did not enhance R123 accumulation, which revealed PG did not allosterically regulate Dox transporter, as shown in Figure 5A. Subsequently, PG-priming showed additional 50–70% of R123 accumulation for long-term priming (12 h). Moreover, the enhancing R123 could be attenuated by autophagy inhibitor, as shown in Figure 5B. This result indicated that PG-priming either enhanced Dox importer expressions or reduced exporter expressions. When checking Dox transporter levels, however, the importer (Oct-6) was not significantly decreased, and exporters (MDR-1 and ABCG2) slightly increased in OECM1 and decreased in SAS and FaDu, as shown in Figure 6. This result was not associated with previous results. It might be postulated as an indication of an unknown but important mechanism of Dox transport.

### 3.4. ER Stress and Energy Deprivation Analysis in PG-Priming OSCC Cells

**Figure 7:**
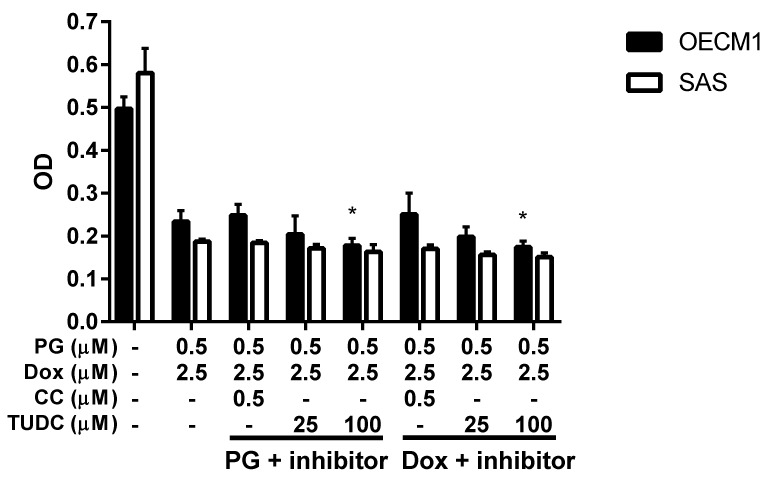
**Cell viability change when PG/Dox combined with ER stress and energy deprivation inhibitors.** OSCC cells were treated with PG + inhibitor/Dox or PG/Dox + inhibitor and were analyzed for cell viability. The inhibitors contained tauroursodeoxycholic acid (TUDC) and dorsomorphin (compound C, CC). The results were represented as mean ± SD from three individual experiments. * *p* < 0.05 compared with PG/Dox.

PG could activate autophagy of OSCC cells as proven by the previous section and literature [48]. In this final part, the aim was to find the trigger of PG-induced autophagy in OSCC cells. As we noted, the two known triggers of autophagy are ER stress (unfolded protein response) and energy deprivation, which may be involved in the PG-priming reaction. ER stress was determined by adding ER stress inhibitor TUDC while energy deprivation was blocked by addition of AMPK specific inhibitor CC. When we combined TUDC or CC with PG and Dox treatment, cell viability of three OSCC cells lines were not significantly changed, as shown in Figure 7, (data of FaDu not shown). This result further postulated that PG-induced autophagy and Dox flux increase were not caused by ER stress and energy deprivation.

## 4. Discussion

The present study is the first demonstration of PG-enhanced Dox influx by activating autophagy in OSCC cells. Based on the Dox flux experiments, the enhancing mechanism of Dox influx was neither related to known importers nor exporters. PG could be a potential adjuvant for Dox treatment. Also, this study excluded the characteristics of known autophagy-triggers in PG-induced autophagy, which posed a new site for autophagy triggering mechanisms. In this work, we first report about the autophagy-activating property of Dox, as this activity was known to be inhibited by the intrinsic activation of autophagy.

Due to the non-tissue specific nature and high-cardiotoxicity, several studies have tried to discover natural compounds that could synergistically potentiate the efficacy of Dox without elevating normal cell toxicity. In a previous report, gambogic acid, a xanthonoid from *Garcinia hanburyi*, sensitized ovarian cancer cells toward Dox through accumulation of ROS [53]. Nitidine chloride, an alkaloid, synergized Dox cytotoxicity in breast cancer cell through PI3K/Akt signaling pathway [54]. Gingerol synergized Dox against liver cancer cells, leading to G2/M arrest [55]. Not only pure compounds; phenolic extract of flaxseed oil also promoted Dox efficacy against breast cancer cells [56]. Evodiamine, a major element of *Evodiae fructus*, reversed chemoresistance in multi-drug resistant breast cancer cells through the Ras/MEK/ERK signaling pathway [57]. Additionally, neferine could combat Dox resistance through ROS accumulation and Fas signaling pathway in lung cancer [58]. In gastric cancer, curcumin and formononetin posed different mechanisms to enhance Dox cytotoxicity [59,60]. These natural compounds exhibit potentiation for main applications in cancer treatment, and also play a supporting role in Dox regimen to overcome the limitation of Dox usage [61,62,63,64,65,66].

The first aim of this study was to explore the synergistic effect within PG/Dox regimen. PG combined with current chemotherapy agents was studied in breast cancer and found that PG could facilitate paclitaxel sensitivity in triple-negative human breast carcinoma cells via down-regulating survivin expression, an anti-apoptotic protein that acts as a caspase inhibitor [67,68]. Our results demonstrated new evidence that PG acts as an adjuvant with conventional chemotherapeutic drugs, such as paclitaxel and Dox as well.

The synergism of natural compounds with Dox could be found in priming fashion, nevertheless the co-treatment is addressed in main efforts [53,54,55,56,57,58,59,60]. While priming with CDK inhibitor in triple-negative breast cancer cell MDA-MB-231, Dox-induced DNA double-strand break would be activated and resulted in cytotoxic enhancement [69]. Cyclophosphamide, a conventional chemotherapeutic drug that acts as an intercalator of DNA, could increase HER2-targeted liposomal Dox accumulation in breast cancer cells [70]. An in vivo study focused on Dox efficacy after mitomycin C and carboplatin (two conventional chemotherapeutic drugs) pretreatment in human metastatic breast cancer-bearing mice. The results showed inhibition growth of xenografted tumors and reducing expressions of p-glycoprotein [71]. In our study, we showed that PG potentiated Dox cytotoxicity only in a pretreatment fashion (as a PG-priming effect), which was the first report of natural compound that primed cancer cells to be sensitized with Dox, and posed the potential of PG as an adjuvant using Dox as a chemotherapeutic agent. The clinical application of PG-priming might provide a great clue for reducing the dosage of Dox and dampening the side effects of Dox.

Due to red fluorescent nature [72], we preliminarily examined PG influx into OSCC cells. The data showed that PG could enter OSCC cells within 1 h and reached saturation after 1.5 h exposure (data not shown). When OSCC cells were primed with PG for 1 h, R123 fluorescent intensity did not significantly accumulate. This gave clear insight into PG action that did not allosterically modulate Dox importer or exporter activity. A previous study has also indicated that PG was not the substrate of multidrug resistance-related protein including MDR-1, BCRP (ABCG2), and MRP2 [73]. Again, our study confirmed that Dox efflux protein was not allosterically activated by PG-priming and further exposed that PG did not allosterically mediate Oct-6 activity.

By R123 accumulation assay, PG did not allosterically control Dox transporter activity but affected transporter expression in long-term priming, as shown in Figure 5. To our best knowledge, the Dox uptake of cells via Oct-6 and excretion of Dox by MDR-1 and ABCG2 have been reported [3,74]. In our study, Dox influx significantly increased in PG-priming for 12 h, which hypothesized that Oct-6 might be up-regulated or MDR-1/ABCG2 down-regulated. However, Oct-6 was down-regulated after PG/Dox treatments in Western blotting. Furthermore, expression levels of MDR-1 and ABCG2 did not show significant reductions. These results proposed a new Dox flux mechanism that needs to be further investigated.

PG was known as apoptotic and autophagic inducer in previous studies [47,75]. According to a recent study, PG induced apoptosis via inhibiting Bcl-2, activating Bak/Bax, intercalating DNA leading to suppress the cell cycle [75]. However, the action of PG-induced autophagy has not been fully explored yet. Remarkably, autophagy was triggered by stresses, such as ER stress (unfolded protein response), nutrient deprivation, and oxidative stress [76,77,78,79]. Hence, these cellular stresses would be the trigger clue of PG-induced autophagy. However, our test exposed that using ER stress inhibitor (TUDC) and nutrient deprivation inhibitor (CC) could not restore the cell viability of PG/Dox. Also, our data showed that PG did not elevate ROS level within OSCC cells (data not shown). All-known causes of autophagy, including ER stress, nutrient deprivation, and oxidative stress, were excluded from the trigger clue of PG-induced autophagy in this study, which the new potential mechanism of autophagy activation necessitates to be further elucidated.

During the screening of an autophagic marker, we also found that Dox-induced autophagy in both OECM1 and FaDu cells. It is well known that Dox was an apoptotic inducer via inhibiting cell cycle and producing ROS [80]. The potentiated role of autophagy in Dox treatment is focused on the activation of autophagy to ameliorate cardiotoxicity, and consequently inhibiting autophagy could promote Dox sensitivity in cancer cells [81,82,83,84]. The autophagy-activating features of Dox suggested the unclear field of Dox action. In the result of autophagy inhibitor recovery assay, autophagic inhibitors could not recover Dox-induced cell death, which implies that Dox-induced autophagy might not be solely involved in Dox-induced cell death, as shown in Figure 3.

**Figure 8:**
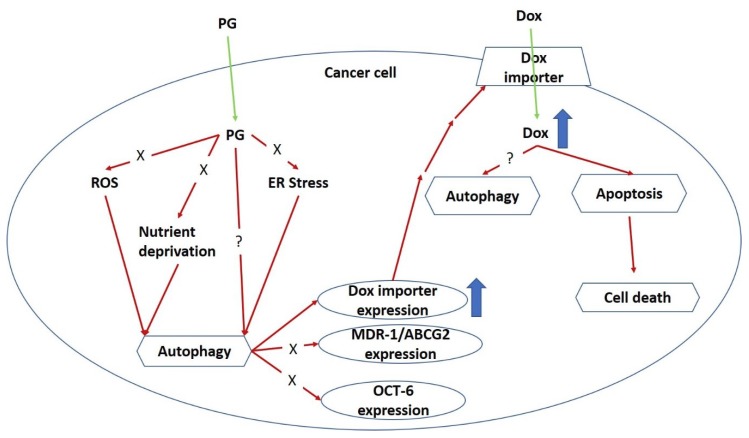
Potential mechanism of PG-priming doxorubicin cytotoxicity enhancement. “X” indicates not according to this mechanism. “?” illustrates still unknown. ER, endoplasmic reticulum; ROS, reactive oxygen species.

Collectively, a model for the mechanical action of PG-priming autophagy potentiated Dox influx is proposed, as shown in Figure 8. When PG entered OSCC cells, autophagy which was irrelevant to nutrient deprivation, ER stress, and ROS, was activated. Subsequently, PG-induced autophagy could up-regulate Dox importer expression and in terms translocated to cell membrane, and consequently led to the enhancement of Dox influx resulting in cell death.

## 5. Conclusions

The present study firstly demonstrated the potential of PG as an adjuvant for Dox treatment in OSCC cells. PG-induced autophagy was not associated with ER stress, nutrient deprivation, and oxidative stress. Also, the enhancement of Dox influx triggered by PG-primed autophagy did not induce via Dox transporter, such as MDR-1, ABCG2, and OCT-6. The potential mechanism of PG-priming remains unclear and would be a further challenge for PG and Dox investigation.

## Figures and Tables

**Table 1:** Antibodies used in this study.

Protein	Host	Source	RRID	MW (kDa)	Dilution
MDR-1	Human	Mouse	AB_2565004	180	1:200
PARP1	Human	Mouse	AB_1127036	116	1:200
ABCG2	Human	Mouse	AB_629007	80	1:200
OCT-6	Human	Mouse	AB_10989254	46	1:200
GAPDH	Human	Mouse	AB_1124759	37	1:1000
Caspase3	Human	Mouse	AB_1119997	32	1:200
LC3 I/II	Human	Mouse	AB_2137722	15/18	1:200
HRP-conjugated 2nd Ab	Mouse	Goat	AB_92635		1:5000

MW, molecular weight; RRID, Research Resource Identifiers.
